# Spongy Scalp Swelling in a Middle-Aged Female: A Case Report of Lipedematous Scalp

**DOI:** 10.7759/cureus.55532

**Published:** 2024-03-04

**Authors:** Shivani Vasisht, Riti Bhatia, Arvind Kumar

**Affiliations:** 1 Dermatology, All India Institute of Medical Sciences, Rishikesh, Rishikesh, IND; 2 Pathology and Laboratory Medicine, All India Institute of Medical Sciences, Rishikesh, Rishikesh, IND

**Keywords:** dysesthesia, lipedematous alopecia, scalp swelling, spongy scalp syndrome, lipedematous scalp

## Abstract

Lipedematous scalp is a rare cutaneous disorder, characterized by subtle but conspicuous scalp swelling, usually associated with dysesthesia. The chronic recalcitrant nature of this condition can be extremely debilitating for the patient. We report a case of boggy scalp swelling and dysesthesia in a 37‑year‑old female present for five years. Magnetic resonance imaging (MRI) brain showed thickening of subcutaneous tissue of the scalp. Histopathological examination revealed thickened and edematous subcutaneous tissue, reaching up to the upper dermis. A diagnosis of lipedematous scalp was made. The patient was reassured about the benign nature of the disease and given symptomatic treatment for dysesthesia. Herein we discuss the approach to a case of boggy dysesthetic scalp swelling and the available treatment options.

## Introduction

Lipedematous scalp, a cutaneous condition of unknown etiology was first described by Cornbleet in 1935 [1]. It presents as a boggy scalp swelling, commonly palpated over the vertex and occiput. This condition is seen more commonly in females. If it presents along with alopecia or short hair, it is known as lipedematous alopecia [2]. It may occur at any age, with patients ranging from 10 to 77 years of age in various case reports [3]. Most patients are asymptomatic, but some may complain of itch, pain, paresthesia, and headache. Here, we report the case of a middle-aged female who presented with painful scalp swelling as her only initial symptom.

## Case presentation

**Figure 1:**
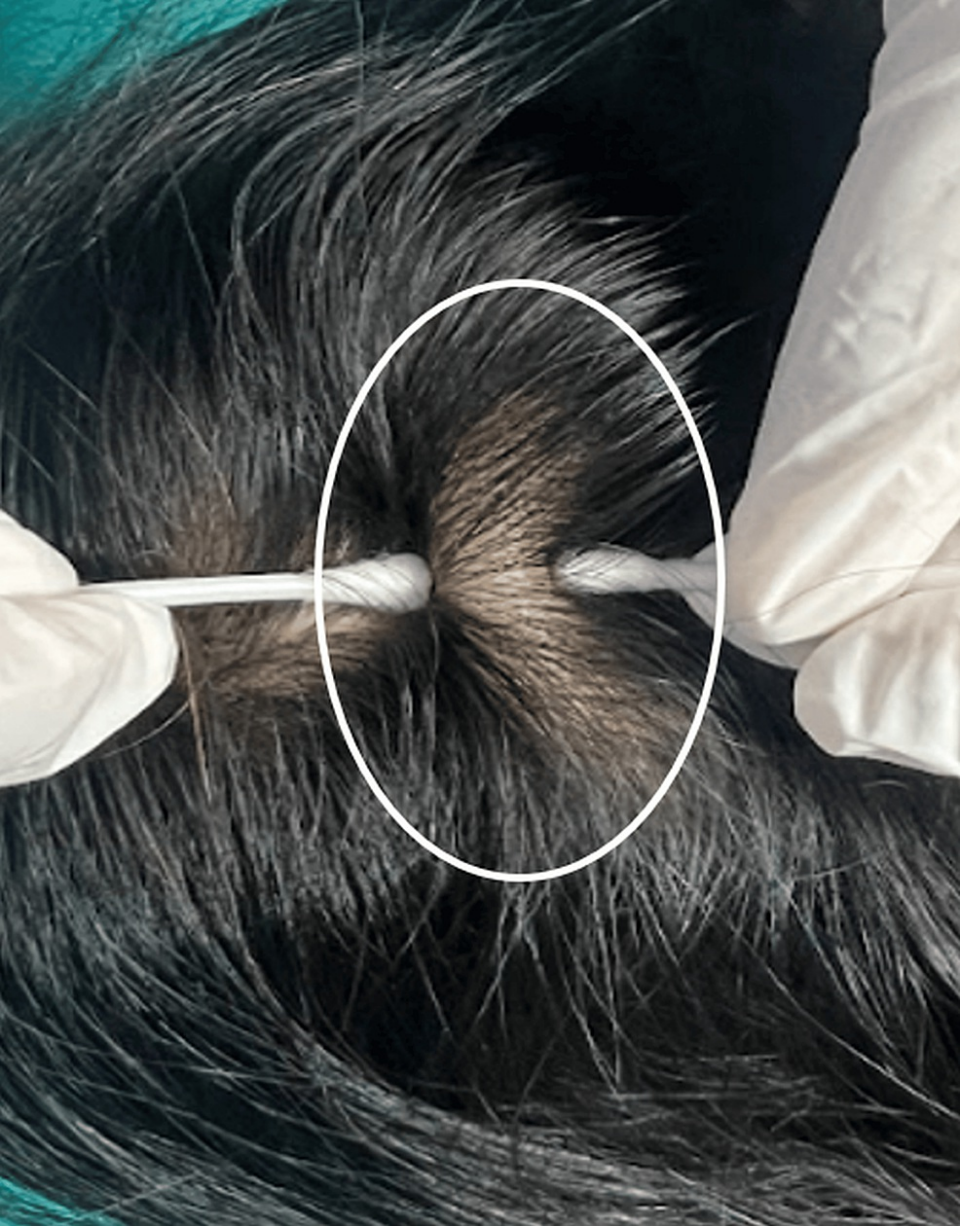
Thickening and cotton-like swelling of the scalp without any hair abnormalities.

A 37‑year‑old female presented to the outpatient clinic of dermatology with the chief complaint of a soft and boggy scalp swelling for five years. It was insidious in onset, initially involving the vertex, and gradually increased in size. The patient also complained of dysesthesia for the last two years. There was no history of trauma at the involved site. She was a known diabetic for 5 years, which was well controlled on metformin. Examination revealed a diffuse, soft, spongy swelling of the scalp, over the vertex and occipital region (Figure 1).

**Figure 2:**
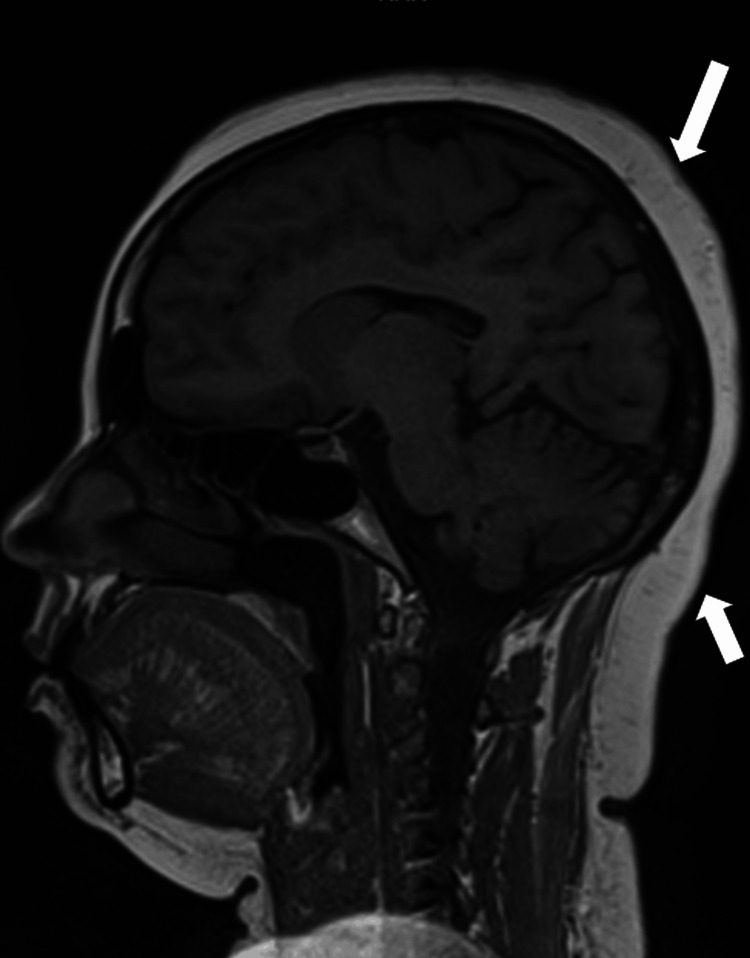
MRI brain (T1) showing scalp thickness of upto 12mm over vertex and occiput (arrows), with normal cortical findings. MRI: Magnetic resonance imaging

The swelling was better palpated than seen and revealed fluctuation of the swollen area. There was no evidence of alopecia, scaling, or erythema. Trichoscopy revealed no abnormality. The hair pull test was negative. The routine blood and biochemistry tests were within normal limits. The antinuclear antibody was negative. MRI brain revealed diffuse thickening of subcutaneous tissue of the scalp of up to 12mm, with prominence over the vertex and occiput. Brain parenchyma appeared normal (Figure 2).

**Figure 3:**
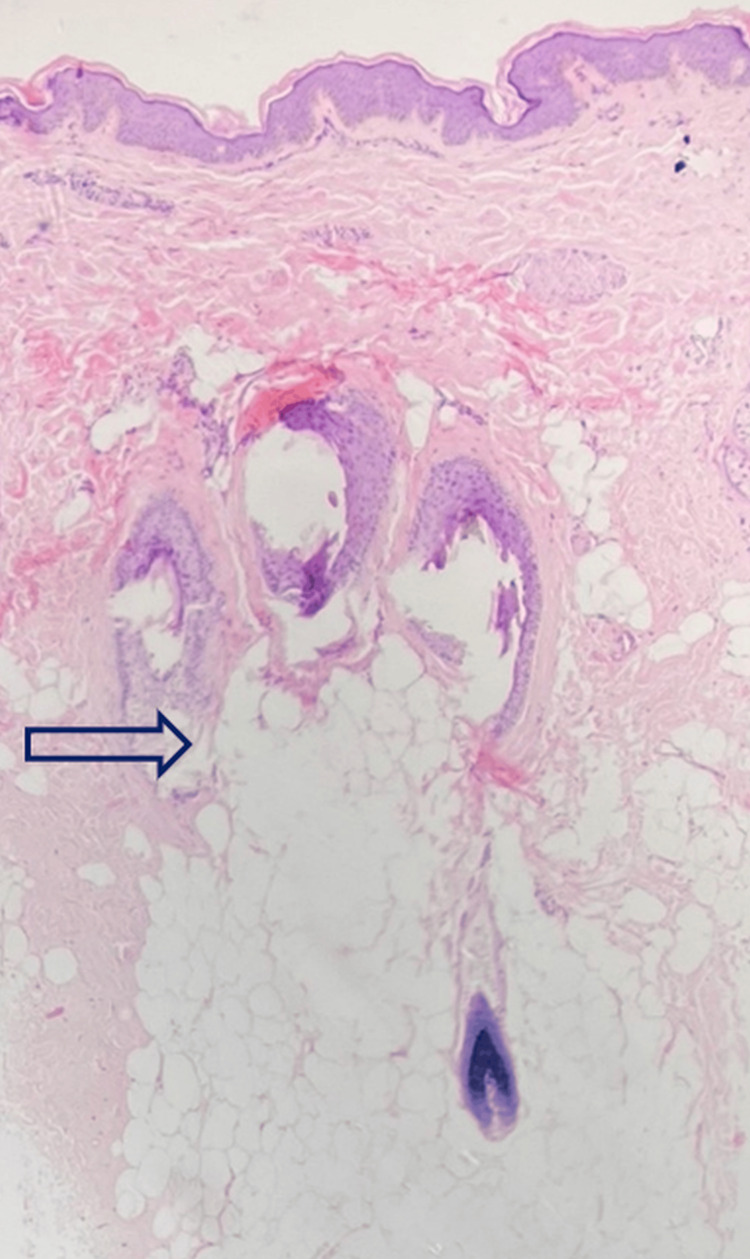
Subcutaneous fat tissue thickening encroaching into dermis (H&E, 4x). H&E: hematoxylin and eosin stain

Following this, a scalp biopsy was done, and histopathology showed thickened and edematous subcutaneous fat composed of mature adipocytes, which appeared to encroach into the dermis (Figure 3).

**Figure 4:**
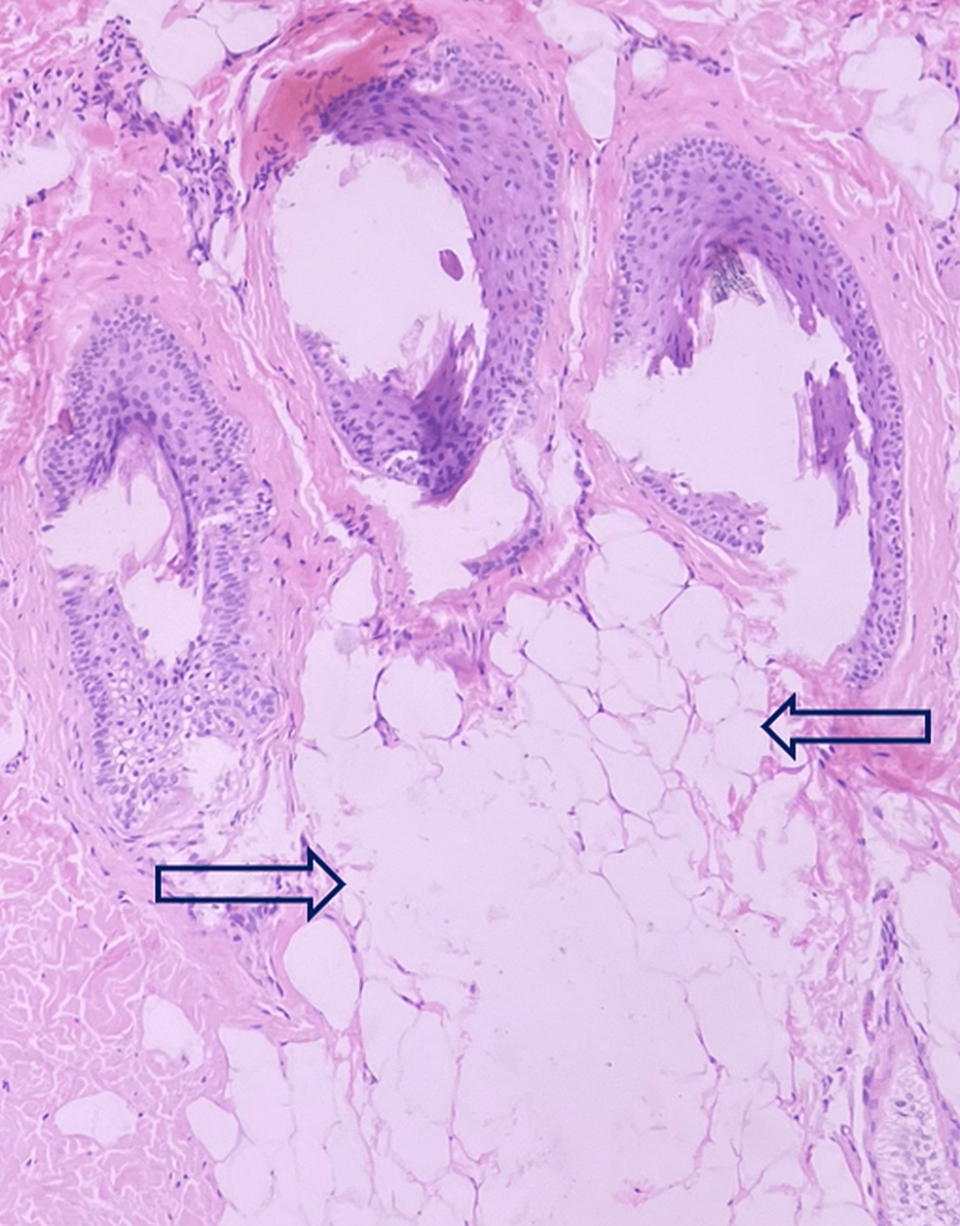
Disruption of adipocyte architecture (arrows) with mild focal perivascular lympho-histiocytic infiltrates (H&E, 10x). H&E: hematoxylin and eosin stain

There was disruption of adipocyte architecture with mild focal perivascular lympho-histiocytic infiltrates (Figure 4).

Based on these findings, a diagnosis of lipedematous scalp was made. The patient was reassured about benign nature of the disease and was given symptomatic treatment in the form of analgesics for dysesthesia. She has been following up in the clinic with no change in her condition and is currently being treated for diabetes mellitus.

## Discussion

Lipedematous scalp was first described in a 44-year-old woman whose scalp showed a cotton-like consistency on palpation [1]. Thereafter in 1961, Coskey et al. gave the term lipedematous alopecia for a disorder presenting as short hair unable to grow beyond 2 cm, overlying a boggy scalp swelling [2]. Recently Müller et al. gave the term “localized lipomatosis of the scalp with or without alopecia” for these two conditions and classified them among the group of conventional lipomatosis [4]. Yasar et al. evaluated the clinical and histopathological features of lipedematous scalp and lipedematous alopecia in 31 patients and found no significant difference in the histopathological findings of these two conditions [5]. Therefore, lipedematous scalp and lipedematous alopecia represent a spectrum of the same disorder.

Though the exact etiology remains unknown, one of the proposed pathogenic mechanisms includes increased leptin, a hormone produced by adipocytes, causing fat hyperplasia and displacement of adipocytes into the dermis [4,6]. The metaplasia and displacement of adipose tissue have also been implicated [7]. Hormonal factors may be involved in its pathogenesis, explaining the female preponderance. Khalil et al. reported lipedematous scalp in two sisters, suggesting a possible genetic component to the pathophysiology of this condition [8]. In a case series of 10 Egyptian women, compression due to tight head gear was implicated in disease causation [9]. Symptoms like headache and dysesthesia may be due to the compression of nerves by edema and thickened subcutaneous fat. Rare associations of lipedematous scalp with alopecia areata and intradermal nevus have been reported [10]. Due to similarities in histopathological features of lipedematous alopecia and lupus erythematosus, the two conditions may coexist, as noted by Klinic et al. [11]. Various studies have shown incidental associations including hyperlipidemia, renal failure, skin hyperelasticity, and diabetes mellitus, as seen in our case [12-14].

Clinically, lipedematous scalp is characterized by scalp thickening, that is better palpable than visible. It can be easily pressed down to the bone but returns immediately to the initial shape upon releasing the pressure. In most cases, the vertex and occiput are involved. The disease shows gradual progression over the years. Although it is usually asymptomatic, it may occasionally be associated with diffuse pain, paresthesias, headache, scalp tenderness, or itching. Histopathologically, thickened subcutaneous tissue appears to encroach into the dermis, and distortion of fat architecture is also seen. Scheufler et al. suggested that the primary histopathological finding in the lipedematous scalp is hyperplasia of subcutaneous tissue, which was noted in our case also [15]. Other findings like dermal edema, ectatic lymphatics, fragmentation of elastic fibers, and deposition of mucin in the dermis are variably seen [5]. The differential diagnoses include cutis verticis gyrata and encephalocraniocutaneous lipomatosis. The absence of gyri, sulci, and herniation helped us exclude these diagnoses. Radiological imaging modalities including ultrasound, computed tomography (CT), and MRI, as in our case are helpful ancillary investigations [16]. In another study, the mean scalp thickness on MRI was observed as 5.5 mm, 7.7 mm, and 7.1 mm at the frontal, occipital, and parietal areas respectively [17]. In patients with lipedematous scalp, the scalp thickness ranges from 9.2 to 16 mm, as also seen in our case [13].

Currently, there is no definitive treatment modality for lipedematous scalp. The use of topical and intralesional steroids and hydroxychloroquine has shown poor clinical response [13]. Surgical debulking combined with scalp reduction was done by Yip et al., with no evidence of relapse after 12 months of follow-up [18]. Systemic treatment with 1g/day of mycophenolate mofetil has shown promising but short-term results [19]. Bukhari et al. reported spontaneous resolution after 13 years in a patient with a lipedematous scalp [20].

## Conclusions

In conclusion, the lipedematous scalp is a rare, albeit distinct disorder. Though the clinical findings are subtle and easy to miss, a careful skin examination along with supportive radiological and histopathological features are helpful in making a diagnosis of this chronic debilitating disorder. Given the paucity of reported cases, there is no consensus on treatment guidelines for this condition. Further studies may be required to establish its etiology and possible treatment options.

## References

[1] Cornbleet T (1935). Cutis verticis gyrata? lipoma?. Arch Derm Syphilol.

[2] Coskey RJ, Fosnaugh RP, Fine G (1961). Lipedematous alopecia. Arch Dermatol.

[3] Carrasco‐Zuber JE, Alvarez‐Veliz S, Cataldo‐Cerda K, Gonzalez‐Bombardiere S (2016). Lipedematous scalp: a case report and review of the current literature. J Dtsch Dermatol Ges.

[4] Müller CSL, Niclou M, Vogt T, Pföhler C (2012). Lipedematous diseases of the scalp are not separate entities but part of a spectrum of lipomatous lesions. J Dtsch Dermatol Ges.

[5] Yasar S, Gunes P, Serdar ZA, Tosun I (2011). Clinical and pathological features of 31 cases of lipedematous scalp and lipedematous alopecia. Eur J Dermatol.

[6] Collins S, Kuhn CM, Petro AE, Swick AG, Chrunyk BA, Surwit RS (1996). Role of leptin in fat regulation. Nature.

[7] Chen E, Patel R, Pavlidakey P, Huang CC (2019). Presentation, diagnosis, and management options of lipedematous alopecia. JAAD Case Rep.

[8] Khalil N, Carton J, Fernandez CP, Patel NP (2024). Lipedematous scalp occurring in two female siblings: further evidence for a genetic role. Skin Appendage Disord.

[9] El Darouti MA, Marzouk SA, Mashaly HM (2007). Lipedema and lipedematous alopecia: report of 10 new cases. Eur J Dermatol.

[10] Sahu P, Sangal B, Dayal S, Kumar S (2019). Lipedematous scalp with varied presentations: a case series of four patients. Indian Dermatol Online J.

[11] Kilinc E, Dogan S, Akinci H, Karaduman A (2018). Lipedematous scalp and alopecia: report of two cases with a brief review of literature. Indian J Dermatol.

[12] Martín JM, Monteagudo C, Montesinos E, Guijarro J, Llombart B, Jordá E (2005). Lipedematous scalp and lipedematous alopecia: a clinical and histologic analysis of 3 cases. J Am Acad Dermatol.

[13] Kavak A, Yuceer D, Yildirim U, Baykal C, Sarisoy HT (2008). Lipedematous scalp: a rare entity. J Dermatol.

[14] Bukhari I, Al Mulhim F, Al Hoqail R (2004). Hyperlipidemia and lipedematous scalp. Ann Saudi Med.

[15] Scheufler O, Kania NM, Heinrichs CM, Exner K (2003). Hyperplasia of the subcutaneous adipose tissue is the primary histopathologic abnormality in lipedematous scalp. Am J Dermatopathol.

[16] Safar L, George S (2021). Lipedema and lipedematous scalp: an overview. J Skin Sex Transm Dis.

[17] Peter CV, Jennifer A, Raychaudhury T, Chandrashekhar L, Merilyn S, Gowda S, Shyam G (2014). Lipedematous scalp. Indian J Dermatol Venereol Leprol.

[18] Yip L, Mason G, Pohl M, Sinclair R (2008). Successful surgical management of lipoedematous alopecia. Australas J Dermatol.

[19] Cabrera R, Larrondo J, Whittle C, Castro A, Gosch M (2015). Successful treatment of lipedematous alopecia using mycophenolate mofetil. Acta Derm Venereol.

[20] Bukhari IA, Bagatadah WA (2016). Spontaneous resolution of lipedematous scalp after 13 years. J Dermatol Plast Surg.

